# Transition to Adult Care for Obstructive Sleep Apnea

**DOI:** 10.3390/jcm8122120

**Published:** 2019-12-02

**Authors:** Austin Heffernan, Uzair Malik, Russell Cheng, Shaun Yo, Indra Narang, Clodagh M. Ryan

**Affiliations:** 1 Sleep Research Laboratory, Toronto Rehabilitation Institute, University Health Network, Toronto, ON M5G2A2, Canada; Austin.heffernan@yahoo.ca (A.H.); Uzair.malik@rcsi.ie (U.M.); russellcheng6@gmail.com (R.C.);; 2 Department of Pediatrics, Division of Respirology, University of Toronto, Toronto, ON M5G1X8, Canada; indra.narang@sickkids.ca; 3 Sleep Laboratory, Hospital for Sick Children, Toronto, ON M5G1X8, Canada; 4 Department of Medicine, Division of Respirology, University of Toronto, Toronto, ON M5G2N2, Canada

**Keywords:** obstructive sleep apnea, healthcare transition, transition to adult care, young adults, adolescents, sleep medicine

## Abstract

Obstructive sleep apnea may occur throughout the lifespan, with peak occurrences in early childhood and during middle and older age. Onset in childhood is overwhelmingly due to adeno-tonsillar hypertrophy, while in adulthood, contributors include risk factors, such as obesity, male sex, and aging. More recently, there has been a precipitous increase in the prevalence of obstructive sleep apnea in youth. Drivers of this phenomenon include both increasing obesity and the survival of children with complex medical conditions into adulthood. Appropriate treatment and long-term management of obstructive sleep apnea is critical to ensure that these youth maintain well-being unfettered by secondary comorbidities. To this end, patient engagement and seamless transition of care from pediatric to adult health care systems is of paramount importance. To date, this is an unacknowledged and unmet need in most sleep programs. This article highlights the need for guideline-driven sleep disorder transition processes and illustrates the authors’ experience with the development of a program for sleep apnea.

## 1. Introduction

For the individual, milestones represent significant key events that may be instrumental in defining or shaping their future. In children with chronic medical conditions, the transfer of their care from the pediatric to adult medical systems represents a significant milestone. This transfer of care to adult health care services typically occurs between the ages of 17 and 19 years and is contingent on either the jurisdiction and/or the patient. While the transfer of care is a one-time event, the transition process from the pediatric to adult health care system is a longitudinal process that occurs in advance of and in conjunction with the transfer. The importance of an organized transition process cannot be underscored. Evidence abounds demonstrating detriments in health and poorer health outcomes for adolescents as they negotiate their life journey from adolescent to adulthood alone [1]. Normal adolescent brain development continues into the early twenties and explains the behavioral hallmarks of increased risk-taking, parent conflict, and greater reliance on peer approval [2]. Non-adherence to medical treatments is a common challenge among adolescents. The patterns of behavior established during adolescence, including the impact of non-adherence with medical treatments, may adversely affect long-term health and quality of life. The absence of a coordinated transition process may lead to gaps in clinical care and potentiate poorer outcomes. With a few exceptions (e.g., transplant, congenital heart disease), these programs do not exist. Some chronic health illnesses, such as hemophilia, cystic fibrosis, congenital heart disease, and diabetes mellitus, have well organized transition processes with clear guidelines in place [3]. However, the majority of other disease programs have little experience or guidance in this domain. This holds true for the field of sleep medicine and in particular, obstructive sleep apnea (OSA). 

This review was performed to evaluate the current knowledge on transition to adult care for young adults with OSA. A literature search of English language articles, reviews, and case reports from 1974 to 2018 was performed in MEDLINE, EMBASE, CINAHL, PsycINFO, and Cochrane databases. As no studies or articles were identified, a narrative review was performed to inform physicians and outline a proposed transition process that could be implemented by other programs. 

## 2. What is Pediatric to Adult Health Care Transition?

Continuity of care for adolescents requires effective transition from pediatric to adult health care. Transition is defined as a process of empowerment that addresses the medical, psychosocial, and educational needs of adolescents with chronic conditions as they move to the adult healthcare system [4,5]. The transition process is not without its challenges and barriers. These include the anxiety associated with both the parents giving up healthcare responsibility and adolescents becoming accountable for their own care [4,6,7]. Adolescents may struggle with leaving their pediatric healthcare team, in particular if there has been a long-standing relationship, and have difficulty with the formation of a strong relationship with the adult provider [4,8,9]. Pediatric clinicians are also hesitant to relinquish their care to an adult healthcare provider due to patient connection and lack of faith in adult care [4,9]. The latter may be justified as adult physicians often lack confidence in treating childhood diseases and in dealing with the emotional impact that childhood diseases have on adolescents [4,6,8,9]. Consequently, it is difficult to find adult healthcare providers who have expertise with the adolescent patient cohort [7,9,10]. When a provider is located, other issues arise, such as communication, coordination, lack of accompanying medical documents, and different practice styles between pediatric and adult healthcare clinicians [8,11]. Other major obstacles faced by transition programs include the integration of medical technology, and inadequacy of institutional and family support [8,9]. 

Therefore, transitions in health care need to be individualized, coordinated, and a collaborative process between youth, caregivers, and pediatric and adult health care providers, spanning both adolescence and adulthood [12,13]. This is emphasized by disease groups that lack a transition program, reporting poor health outcomes that may be attributed to the transfer of care itself [14].

## 3. Obstructive Sleep Apnea

OSA is a chronic form of sleep disordered breathing characterized by snoring and recurrent obstruction (apneas) of the upper airway, which disrupts normal ventilation during sleep and predisposes to sleep fragmentation and intermittent hypoxia. Historically, pediatric OSA has a prevalence rate of 2% to 4% [15], usually between 2 and 8 years of age coinciding with adenotonsillar hypertrophy where an adenotonsillectomy is typically curative [16]. OSA has a male predominance in the youth age group [17]. Incidence is substantial, as 4% to 10% of children from middle childhood develop OSA in late adolescence [17,18]. In the last 10 years, there has been a paradigm shift in pediatric OSA, with a marked increase in youth diagnosed with persistent, severe OSA [15]. This increase can be attributed to (1) the current obesity epidemic as 16% of youth in the Western world are obese [19], 25% to 60% of whom will have obesity-related OSA [20], and most will remain obese as only 1.6% of youth revert to non-obesity in adulthood [21]. The increase in OSA is also due to (2) advances in medical therapies and technology that have increased the life expectancy of children with medical complexity (e.g., congenital heart disease) and (3) improved diagnostics and increased awareness. Therefore, there are increased volumes of youth with OSA who need to have care transferred from pediatric to adult health care providers. 

## 4. OSA and Treatments

There are multiple treatments for OSA, but the mainstay of therapy for youth with severe persistent disease is positive airway pressure (PAP), which delivers pressurized air via nasal or oronasal interfaces to distend the upper airway and ameliorate OSA. PAP use in the pediatric population at home has increased three-fold in the past decade [22]. Although PAP is the most efficacious therapy for OSA, less than 50% of youth and adults will be adherent to PAP [23,24]. Lack of adherence to PAP is multifactorial, including difficulty adapting to medical technology, lack of support from health care team [25,26] and/or caregivers, discomfort of mask, and lack of perceived benefits of PAP [27]. Youth may be affected emotionally, have low self-esteem, and become negatively impacted socially and academically [28,29].

Additional OSA therapies include but are not limited to positional therapy, oral dental appliances, and hypoglossal nerve stimulation. Positional therapy improves the apnea-hypopnea index in adults with OSA [30], but due to a lack of evidence supporting its efficacy in children [31], most transitioning adolescents are not on positional therapy. Similarly, there are limited clinical studies and experience to support or refute the use of dental appliances in the pediatric population [32]. Novel therapeutic approaches, such as hypoglossal nerve stimulation, are unavailable to most patients and evidence is limited with respect to both short and long efficacy [33]. The longitudinal follow-up of youth will facilitate ongoing discussions regarding treatment of OSA and the opportunity to trial new and advanced modalities of treatment as they arise. 

## 5. Why is a Transition Process Necessary for Adolescents with OSA? 

Recent data on the OSA disease trajectory from childhood into young adulthood demonstrated that complete resolution may occur in a proportion of children but that adolescent OSA was more likely to persist [34]. An OSA transition process is essential because OSA represents a significant health burden and more than 80% of adults are undiagnosed and/or undertreated for many years [35,36]. In fact, the American Academy of Sleep Medicine recently reported that OSA is a ‘hidden health crisis’ [36]. There is a wealth of evidence showing longstanding untreated OSA in adults impacts nearly all key indicators of health, including cardiovascular [37], cerebrovascular [38], metabolic morbidities [39], work performance and productivity, daily functioning, quality of life (QOL), and mental health, particularly anxiety and depression [40].

There is emerging evidence that even in youth, OSA is an independent risk factor for cardiovascular and metabolic risk, all cancer incidence and mortality, fatty liver disease [41,42,43], adverse outcomes in psychosocial functioning, increased anxiety and depression [40], lower academic performance, and lower QOL [44,45]. Moreover, there is a three to seven times increase in the risk of motor vehicle crashes related to daytime sleepiness associated with untreated OSA [35]. Youth and adults treated with PAP have demonstrated improvements in cardiovascular and metabolic risk profile, improved quality of life, decreased anxiety/depression rates, and reduced risk of accidents and injury [39,46,47]. 

The economic burden of OSA is also substantial as undiagnosed and/or undertreated OSA costs $150 billion per year in the USA due to direct and indirect health costs, including loss of work productivity, accidents, and absenteeism [36,48]. Treatment of OSA is associated with significant reductions in both direct health care costs and health care utilization [49,50,51].

There are currently no described OSA pediatric to adult transition programs despite the occurrence of pediatric and adult sleep programs both in community settings and academic centers across Canada and internationally. Although the literature is limited on the long-term successes of effective transitions, research evaluating structured transition interventions in chronic disease groups, e.g., diabetes [52], cystic fibrosis [53], and juvenile arthritis [54], endorse positive disease and psychosocial outcomes short term, in the year following transition [19]. However, successful transition care models for single diseases, such as diabetes, may not be applicable for youth with OSA. Youth with OSA are a unique cohort of patients as they represent marked heterogeneity in underlying diseases and require advanced medical technology every night while sleeping. 

## 6. Transition Programs for Youth with OSA

Youth with OSA who transition to adult health care should be followed up regularly. This will involve assessments of OSA and PAP requirements using overnight polysomnograms, monitoring of PAP adherence, prescription for new PAP devices, as well as continuing education around OSA and PAP therapy. Early adoption of PAP therapy in youth requires a multidimensional approach and is associated with improved patterns of adherence [55]. Further, follow-up allows patient access to emerging novel pharmacological agents as alternatives to PAP [56]. Trained adult health care providers can also anticipate and monitor disease outcomes, which may manifest earlier in these youth, particularly as obesity and OSA may synergistically magnify the risk of adverse health outcomes [57]. Regular monitoring for adverse disease outcomes allows for targeted therapeutic interventions in high-risk patients in a timely manner. Finally, recognition and treatment of co-existing diseases, such as depression, that may modify PAP adherence will require follow-up and ongoing support. An essential first step to optimize care for youth with OSA and to acquire knowledge around the trajectory of OSA disease is to ensure access to adult sleep services where youth can receive age and developmentally appropriate care through well-structured transition programs.

## 7. Establishing a Transition Program for Youth with OSA

In 2013, the sleep team at the Hospital for Sick Children (SickKids), Toronto, Canada conducted an audit (unpublished, results from author) showing only 38% of youth on PAP attended their first adult sleep clinic at Toronto General Hospital (TGH). Those that did attend had clear gaps in knowledge, specifically: (1) Reason for clinic, (2) OSA diagnosis, (3) importance of continuing PAP use, and (4) need for follow-up. 

In response, and to mitigate potential adverse health outcomes on these youth, a unique hospital-based pediatric to adult transitional integrative care program was developed and implemented by the Hospital for Sick Children (SickKids) and University Health Network-Toronto General Hospital (UHN-TGH) in 2015. The Adolescent Medicine’s Good 2 Go Transition Program at SickKids partnered with the sleep team to support the development of the Sleep Disorders Pediatric Transition (SlePT) program. The SlePT program incorporated recommended guidelines and design principles [1,58,59]. In the SlePT program, youth with OSA requiring PAP are either obese and/or have an underlying medical condition, the majority are ambulatory, and more than 50% demonstrate the ability to function independently. 

## 8. Clinical Transition Program—General Principles 

There are five guiding principles that guide the implementation of our sleep disorder transition program. These principles are implemented in conjunction with the clinical transition algorithm recommended by the American Academy of Pediatrics, American Academy of Family Physician and American College of Physicians [11] designed to aid and direct health care providers (Table 1). 

**Figure 1:**
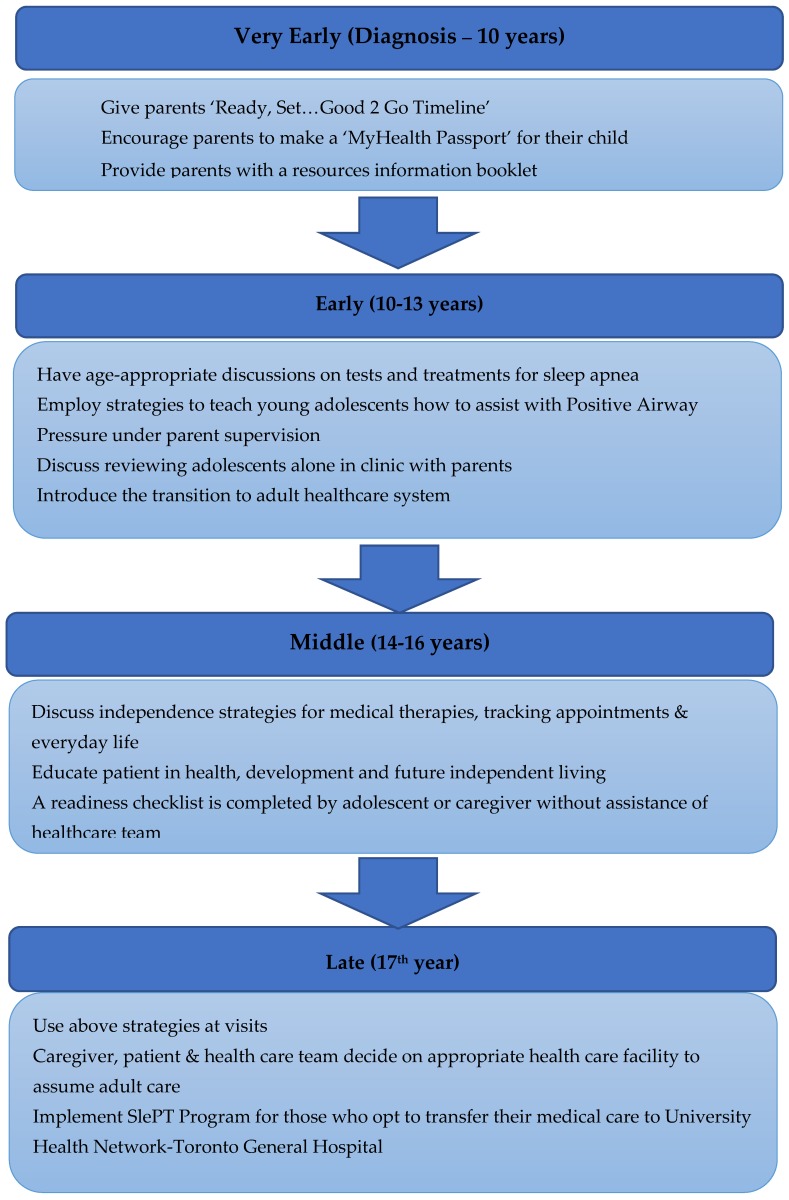
Overview of Transition Process for Sleep Apnea.

**Figure 2:**
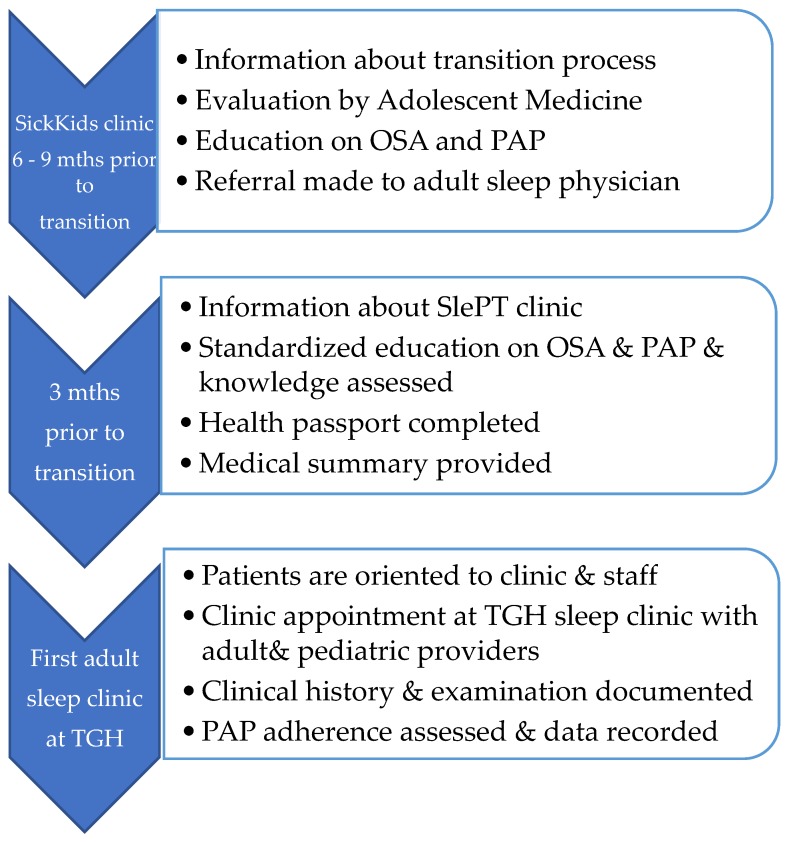
Description of Current SlePT Program.

**Figure 3:**
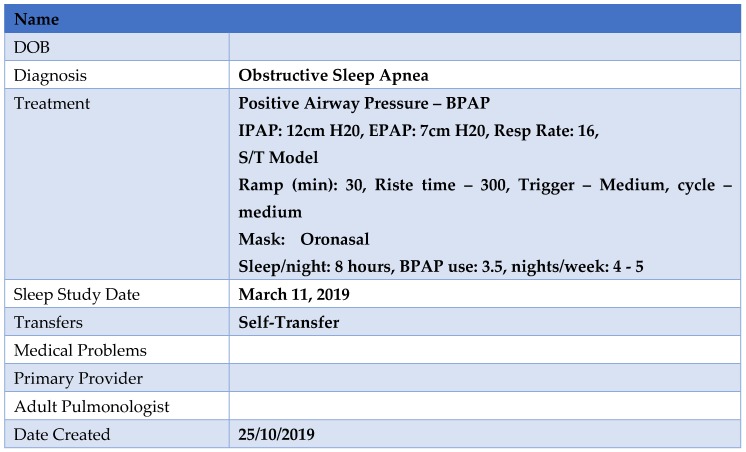
Example of MyHealth Passport.

**Figure 4:**
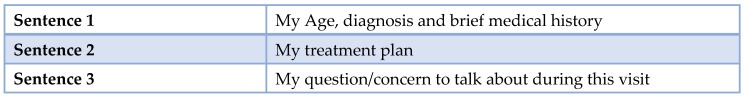
Outline of MyHealth Summary Template.

As part of this process there are age ranges at which specific actionable goals should be undertaken, with the determination of special needs and a cohesive plan for the chronic disease management prior to the transfer (Figure 1). These principles have been employed by the Sleep Disorders Clinic at the Hospital for Sick Children (SickKids) (Figure 2) and include the completion of ‘MyHealth Passport’ [60] (Figure 3) and ‘MyHealth Summary’ [61] (Figure 4). 

During the final year in the pediatric health care setting, all patients for transition, regardless of medical history, are discussed at the pediatric sleep multi-disciplinary clinical care rounds. At these rounds, pediatric patients are identified as requiring follow-up at an adult sleep clinic, e.g., SlePT clinic. The SlePT clinic will also see young adults enrolled in longitudinal sleep research studies and patients with multiple comorbidities and complex medical disorders who also require follow-up at other specialist clinics at the University Health Network. A decision is made with the caregivers/patients regarding the appropriate health care facility/physician to assume adult care. For those who opt to transfer their medical care to UHN-TGH, the SlePT Program is implemented for transitioning adolescent patients (see Figure 3). For those who wish to follow-up with another adult provider, a transfer letter is sent at the appropriate time-point. 

## 9. Sleep Disorders Pediatric Transition (SlePT) Program

The goals of the SlePT Program are focused around all stakeholders and include a mandate to: (1) Enhance knowledge and empower youth with OSA; (2) introduce to the adult team and orientate to the adult system; (3) improve collaboration, linkages, and communication between the sleep specialists to promote an integrated knowledge of medical information; (4) provide education to the adult health care providers of the unique needs of young adults; and (5) provide a coordinated platform to seamlessly transition adolescents with OSA who require care for additional co-morbid disease at UHN-TGH. 

Within the SlePT program, a standardized protocol is followed as outlined: 

(i) Sleep clinic 6 to 9 months prior to transfer: At this clinic, the patient and caregiver will receive: (1) Information of the goals, timelines, and outline of the SlePT process; (2) a further review by a staff physician of the adolescent medicine team who will address any psychosocial concerns; (3) ongoing teaching and education around OSA; and (4) PAP device education and strategies to optimize adherence. At this visit, a repeat readiness checklist is completed to identify any gaps that need to be addressed prior to transfer. For instance, at this point, the adolescent should know names and doses of all medications, have primary responsibility for administering and re-ordering medications, and be comfortable with their PAP machine [62]. They should also understand their health history and current conditions, appreciate the importance of long and short-term complications, and be aware of the impact of PAP on their health [62]. Medical professionals also confirm the mechanisms of drug coverage as an adult with the adolescent and facilitate application for adult disability benefits, as appropriate. Lastly, administrative tasks are completed at this clinic visit including a referral to the SlePT Clinic. 

(ii) Sleep clinic 3 months prior to transfer: This visit follows a standardized protocol which begins by (1) the patient and caregiver receiving a brochure for the SlePT clinic. It includes the date, time, and location of the SlePT clinic, an introduction to adult-oriented healthcare, information on the responsibility placed on the patient, and answers to frequently asked questions. (2) The patient and caregiver receive standardized education by the sleep multi-disciplinary team about OSA, PAP treatment, and efficacy as well as adverse health consequences of untreated OSA; (3) teaching around the functioning of their PAP device; (4) a health passport with pertinent details of PAP therapy; (5) a medical summary is provided to the patient outlining their diagnosis and treatment(s); and (6) a final review by pediatric staff is completed. The patient is also reminded that Sick Kids will continue as the primary contact until the patient is seen by the adult sleep physician at UHN-TGH.

(iii) SlePT Clinic Visit: The SlePT Clinic is an integral part of the SlePT Program and is held at the adult facility at TGH, with patients seen by both the pediatric and adult sleep teams. The framework of this visit includes: (1) Introduce and orient young adults and/or caregivers to the adult sleep physician, staff, and the clinic; (2) orient the young adult to the adult health care system and clinic; (3) review and update the ‘MyHealth Passport’ and complete the baseline health and sleep questionnaires; (4) there is a verbal and written handover of medical information and care between clinics; and (5) clinical history, examination, and PAP adherence and usage data are recorded. 

(iv) Adult Sleep Follow-Up Clinic: Adult sleep physician continues to follow-up the patients regularly and all subsequent clinic visits occur in the adult sleep clinic at UHN-TGH. 

## 10. Evaluation of a Sleep Disorders Program

While evidence suggests that much is to be gained by the patient and society through the introduction of transition programs, it behooves any program to perform both initial and continuous evaluations. These audits should be aimed at identifying areas for improvement and assessing efficacy and overall impact. A universal challenge exists in identifying and measuring outputs of successful transition programs and subsequently, there is a paucity of evidence-based practices and guidelines. An evaluation should be structured around the current recommendations from the Institute for Healthcare Improvement using the Triple Aim as an evaluative framework [63]. The three domains of the triple aim are: (1) Health of a population (e.g., adherence to care guidelines, disease-specific outcomes, patient-reported QOL, functional status, self-care skills, and process of care; (2) individual experiences of care (satisfaction, enablers, and barriers to care); and (3) cost measures, e.g., gaps in care. Evaluative questionnaires have been developed in different health care systems and for differing disease processes, many of which may not translate internationally or across medical diseases. We have included an example of an assessment program (Table 2), which can be adjusted or expanded for the relevant healthcare system. 

## 11. Limitations 

While striving to perform a comprehensive review of the literature, our review may be potentially biased as it does not provide a critical appraisal of the articles included and is limited to those in the English language. Although there was a paucity of publications on transition care for OSA, it does not rule out the existence of transition programs. Furthermore, our transition program was developed for two academic medical facilities within the Canadian healthcare system. Our model is hospital based and not a fully integrated care model for the patient. While we believe that it could be implemented in other public healthcare settings, we realize that different healthcare funding and care models may preclude the execution of a similar transition model. Lastly, to date, we have not performed an evaluation of our transition model and therefore, its efficacy is uncertain.

## 12. Conclusions 

As a result of the obesity epidemic and the improved survival to adulthood of those with chronic medical illnesses, increased numbers of young adults will have OSA and require ongoing long-term medical follow-up. Appropriate education of adult providers and implementation of transition programs is important to facilitate the ongoing care of this potentially vulnerable group. Our program has only been operational for a few years, but there has been a gradual and sustained increase in those adolescents requiring follow-up care. This paper outlines our transition process for adolescent patients with sleep apnea. Although this transition program was developed for the Canadian health care system, we believe that this model could be applied in other jurisdictions. This may require minor modifications, such as the use of virtual care, and/or the identification and education of a network of community physicians who could provide transition care. As with any new program it is critical that assessments are performed to determine clinical efficacy, efficiency, and effectiveness to identify gaps in care and deficiencies that need to be addressed [13]. We believe that the implementation of sleep disorders transition programs in tandem with an ongoing evaluative framework is important to effectively transition and integrate adolescents into adult health care systems.

## Figures and Tables

**Table 1:** General principles for clinical transition programs.

Guiding Principles	Details
1. Begin Transition Planning Early	Begin at diagnosis or 10 years Provides sufficient time for transition preparation
2. Uninterrupted Healthcare Delivery	Ensure a continuous source of care for adolescent population
3. Comprehensive Involvement	Patient, caregiver(s), family, and interdisciplinary team of healthcare Professionals should be involved in transition process
4. Recognize Differences in Needs	Patients and their caregivers and family may have differing needs Needs must be assessed and addressed regularly Applicable resource: readiness checklists

**Table 2:** Evaluation framework for Sleep Disorders Pediatric Transition (SlePT) Program.

Element of Triple Aim	Measure
**Health of a Population**	
Disease Management and Self-Efficacy	STARx Transition Readiness Questionnaire for patients in the pediatric and adult health-care settings [64] STARx Questionnaire -for caregivers
Psychosocial Functioning, Mental Health and Health-Related Quality of Life	Pediatric Quality of Life Inventory (PedsQL) [65] Strengths and Difficulties Questionnaire (SDQ) [66] The Columbia Impairment Scale (C.I.S) [67] CAGE-AID [68]
OSA-Specific Outcomes	Functional Outcomes of Sleep Questionnaire (FOSQ-10) [69] Adherence Barriers to CPAP Questionnaire (ABCQ) [70] Objective PAP adherence
**Individual Experiences of Care**	
Expectations and satisfaction with healthcare Barriers to Care	Health Care Transition Feedback Survey for Youth and Parents [71] Qualitative semi-structured interviews with caregiver and patient before and after transfer of care
**Cost Measures**	
Gaps in Care	Health care utilization questionnaire Clinic attendance Time between last pediatric and first adult visit Missed appointments
